# Factors associated with seasonal influenza and HPV vaccination uptake among different ethnic groups in Arab and Jewish society in Israel

**DOI:** 10.1186/s12939-021-01523-1

**Published:** 2021-09-07

**Authors:** Nour Abed Elhadi Shahbari, Anat Gesser-Edelsburg, Nadav Davidovitch, Shuli Brammli-Greenberg, Rami Grifat, Gustavo S. Mesch

**Affiliations:** 1 School of Public Health, University of Haifa, 199 Aba Khoushy Ave. Mount Carmel, 3498838 Haifa, Israel; 2 School of Public Health, Founding Director of the Health and Risk Communication Research Center, University of Haifa, 199 Aba Khoushy Ave. Mount Carmel, 3498838 Haifa, Israel; 3 Department of Health Systems Management, Faculty of Health Sciences, Ben Gurion University of the Negev, 84105 Beer Sheva, Israel; 4 Braun School of Public Health and Community Medicine, The Hebrew University of Jerusalem, P.O. Box 12272, 9112102 Jerusalem, Israel; 5 Ziv Medical Center, 1 Derech HaRambam, 13100 Safed, Israel; 6 Department of Sociology, University of Haifa, 199 Aba Khoushy Ave. Mount Carmel, 3498838 Haifa, Israel

**Keywords:** Vaccinations, Seasonal influenza, Human papillomavirus (HPV), Decision-making, Arab population of Israel, Ethnicity, Trust, Health orientation, Social marketing, School-located vaccination program

## Abstract

**Background:**

Parents in the Arab population of Israel are known to be “pro-vaccination” and vaccinate their children at higher rates than the Jewish population, specifically against human papilloma virus (HPV) and seasonal influenza.

**Objectives:**

This study seeks to identify and compare variables associated with mothers’ uptake of two vaccinations, influenza and HPV, among different subgroups in Arab and Jewish society in Israel.

**Methods:**

A cross-sectional study of the entire spectrum of the Israeli population was conducted using a stratified sample of Jewish mothers (*n* = 159) and Arab mothers (*n* = 534) from different subgroups: Muslim, Christian, Druse and Northern Bedouins. From March 30, 2019 through October 20, 2019, questionnaires were distributed manually to eighth grade pupils (13–14 years old) who had younger siblings in second (7–8 years old) or third (8–9 years old) grades.

**Results:**

Arab mothers exhibited a higher rate of uptake for both vaccinations (*p* < .0001, HPV – 90%; influenza – 62%) than Jewish mothers (*p* = 0.0014, HPV – 46%; influenza – 34%). Furthermore, results showed that HPV vaccination uptake is significantly higher than seasonal influenza vaccination uptake in both populations. Examination of the different ethnic subgroups revealed differences in vaccination uptake. For both vaccinations, the Northern Bedouins exhibited the highest uptake rate of all the Arab subgroups (74%), followed by the Druse (74%) and Muslim groups (60%). The Christian Arab group exhibited the lowest uptake rate (46%). Moreover, the uptake rate among secular Jewish mothers was lower than in any of the Arab groups (38%), though higher than among religious/traditional Jewish mothers, who exhibited the lowest uptake rate (26%). A comparison of the variables associated with mothers’ vaccination uptake revealed differences between the ethnic subgroups.

Moreover, the findings of the multiple logistic regression revealed the following to be the most significant factors in Arab mothers’ intake of both vaccinations: school-located vaccination and mothers’ perceived risk and perceived trust in the system and in the family physician. These variables are manifested differently in the different ethnic groups.

**Conclusions:**

This research shows that all Arabs cannot be lumped together as one monolithic group in that they exhibit major differences according to religion, education and access to information. Ranking of variables associated with uptake of the two vaccines can provide decision-makers an empirical basis for tailoring appropriate and specific interventions to each subgroup to achieve the highest vaccine uptake rate possible. Media campaigns targeting the Arab population should be segmented to appeal to the various sub-groups according to their viewpoints, needs and health literacy.

**Supplementary Information:**

The online version contains supplementary material available at 10.1186/s12939-021-01523-1.

## Background

The research literature identifies different types of decision-makers in the context of vaccinations: pro-vaccination, hesitant (selective choice of when and for what to vaccinate) and anti-vaccination. Each type is marked by its own considerations and decision-making processes. Most studies point to lower levels of vaccination among minority population groups than among dominant groups [1–4].

Arabs living in Western countries as minority groups tend to vaccinate their children less than the dominant national group [5–9]. Nevertheless, a few studies show a higher vaccination rate among the children of Arab minorities living in Western countries [10].

The low vaccination uptake rate among minority groups in high income countries stems from various reasons. The main ones include lack of trust [11–13]; hostility toward the government [14]; medical staff language barriers and inability to understand patients’ values, norms, language and behavior [6, 15, 16]; opposition to institutional recommendations [17]; inability to integrate into the life of the dominant society [18, 19]; limited knowledge about vaccinations [16, 20]; other social and economic factors, such as high income [21–23] and low educational level [6, 24, 25], both of which increase the chances of vaccination uptake; traditional beliefs [26]; and sex of child in the case of the HPV vaccine [27, 28].

As opposed to the aforementioned Arab minority groups living in Western countries, parents in the Arab population of Israel are known to be “pro-vaccination” and tend to vaccinate their children at higher rates than the Jewish population, specifically against the human papillomavirus (HPV) and seasonal influenza [29].

These two vaccinations were recently introduced into the Israeli schools. The influenza vaccine is given at school both to boost vaccination uptake rates and because influenza is a common infectious disease among children. Moreover, the HPV vaccine can be targeted at school before children become sexually active. In 2013, the HPV vaccine was included as part of the planned routine vaccines given in school to girls in the eighth grade, and was later extended to include all eighth graders (13–14 years old), including boys [30]. According to the Israel Ministry of Health [30], in 2016 the uptake rate for the HPV vaccine in Arab schools reached 84% (96% among the Northern Bedouins), compared to 40% among the Jewish population.

Similarly, in 2016 s grade pupils (7–8 years old) in Israel began receiving the live attenuated seasonal influenza vaccination at school. In 2017, third graders (8–9 years old) were also included in the school-located influenza vaccination program, with some children receiving the first dose of the vaccine and some receiving the second. Beginning in September 2018, fourth graders were also included in the school-located vaccination program, such that during the 2019–2020 influenza season, all pupils in the second to fourth grades were offered one dose of the seasonal influenza vaccine at school [31].

After the seasonal influenza vaccine was introduced to the school-located vaccination program in the 2016–2017 influenza season, the uptake rate for second graders in the Arab schools was 84%, compared to 47% among the Jewish population. The Ministry of Health’s vaccination report for 2019 points to higher vaccination coverage in the Arab schools (81.4%) than in the schools in the Jewish sector (44–54%). The primary reason for not vaccinating children was parental refusal (94%). Children who required a second dose and never received influenza vaccinations in the past were instructed to complete the vaccination at their HMO (Health Maintenance Organization) [32]. It should be noted that uptake of these two vaccinations among the Jewish population is much lower than uptake of other routine school vaccinations, such as MMRV (96%) and Dtap IPV (95%) [33].

Alongside a large body of evidence indicating the effectiveness and safety of the HPV vaccine [34–36], the research literature also reveals a scientific controversy surrounding the safety of this vaccine. Several smaller studies examining the HPV vaccine reported side effects, some relatively minor such as pain at the injection site, fainting and dizziness, and some more serious, such POTS (Postural Orthostatic Tachycardia Syndrome), neurological disturbances (CRPS—complex regional pain syndrome), leg paralysis, autoimmune diseases and sympathetic nervous system deficiencies [37–39]. Barriers to the HPV vaccination are related to taboos in conservative societies prohibiting sexual relations before marriage [40–43]. These fears are common among the Arab population as a whole, and particularly among the Muslim population, as well as among orthodox Jews [44–47].

Moreover, despite studies pointing to the effectiveness of the seasonal influenza vaccine [48–50], some studies report a controversy surrounding its effectiveness [43–46]. Studies have pointed to varying effectiveness according to age group: 54% (age 6–17), 61% (under the age of 5), 70% (6 months-8 years), 73% (2–5 years), 78% (6 months to 7 years). Regarding influenza vaccine efficacy, different research studies have also shown that vaccine efficacy varies by age group: 28% (age 2–5), 59% (6 months-15 years), 60% to 83% (6 months − 7 years), 61% (under the age of 5, age 6–17) and 69.6% (5–17 years) [5, 51, 52].

In 2020 the Arab population of the State of Israel numbered about two million people, constituting 25% of the general population. Of these, 82% are Muslim, 9% are Christian and 9% are Druse. Fifty-three percent of Arab families live in poverty, compared to 14% of Jewish families. Over the years, the educational level of the Arab population has improved, yet the educational gaps between Arabs and Jews remain large. Of Arab women between the ages of 25 and 34, 29% completed 16 or more years of education, compared to 50% of Jewish women in the same age group [53].

Jewish society is divided into several groups: secular (45%), traditional (35%), religious and very religious (16%) and ultra-Orthodox (14%). In this study we examined the traditional group, which is located on a spectrum somewhere between religious and secular [54]. For the most part, traditional Jews observe specific commandments and traditions considered to be clear signs of traditional belief. They do so not necessarily out of strict compliance with Jewish law but rather out of a sense of identification and belonging with the Jewish people or out of a belief that these traditional values must be safeguarded to guarantee the existence of the Jewish people [54].

The Israeli school system is marked by a great deal of segregation. Arabs and Jews do not attend the same schools. Moreover, the very religious and ultra-Orthodox groups attend different schools from the secular and traditional groups and sometimes from each other [55], leading to inequality in education, research and policy. Jews and Arabs also tend to live in different residential areas under separate municipal authorities, pointing to spatial politics and discrepancies between Jews and Palestinians within Israel [56].

In view of the interesting phenomenon of high vaccination rates among the Arab population of Israel, this study focuses on the factors related to decision-making among Arab mothers in Israel regarding these two vaccinations: seasonal influenza and HPV. These two vaccinations were chosen for two reasons: 1) They were recently introduced to the school-located vaccination program. 2) Both are a matter of controversy—regarding safety in the case of the HPV vaccination and regarding effectiveness in the case of the influenza vaccination.

In addition, very few research studies have examined vaccination uptake rates among the various subpopulations in Arab society, with most research tending to consider the Arab population as a single entity. This study seeks to examine these two issues. It investigates the variables influencing vaccination uptake among subgroups in the Arab population (Muslims, Christians, Druse and Northern Bedouins), while comparing vaccination uptake to that of the national Jewish population (secular and religious groups).

The overarching goal of this study is to rank the extent of uptake of these two vaccinations—seasonal influenza and HPV—among the subgroups in Arab society and in Jewish society, from the highest uptake rate to the lowest.

The specific research objectives are as follows:

- To compare vaccination uptake of the HPV and seasonal influenza vaccines in the Arab population to that in the Jewish population.
- To identify and characterize the variables associated with mothers’ uptake of these two vaccinations.
- To compare vaccination uptake of the HPV and seasonal influenza vaccines in the different ethnic subgroups
- To compare the differences between ethnic subgroups for each variable associated with mothers’ vaccination uptake.

## Methods

### Research population

The research population included mothers with children in both of the following two age groups:

1. A child in second or third grade, such that the mothers must decide whether their child should get the seasonal influenza vaccination that was recently introduced to the school-located vaccination program.
2. A child in the eighth grade, such that the mothers must decide whether their child should get the HPV vaccine, also part of the school-located vaccination program.

Mothers who had children both in elementary school and in middle school were included, while mothers who had children in only one of these two age groups were excluded from the study. We chose mothers with two children of different ages in order to compare the mothers’ decision-making with respect to the two different vaccinations. Our rationale in choosing mothers was that commonly mothers are the primary parent in the family when it comes to making decisions about vaccinations [29].

### Sampling method and research procedure

The sample was chosen by means of stratified sampling [57] according to the ethnic subgroups examined. The sampled subgroups were of equal size rather than in accordance with their relative proportion in the population of Israel. Hence, each group had the same number of participants, facilitating group comparisons. After the study was approved by the Ethics Committee of the Faculty of Social Welfare and Health Sciences at the University of Haifa (Approval No. 118/16), participants were recruited by means of stratified heterogeneous sampling [58] at schools in a number of different localities in Israel. During the period March 30, 2019 through October 20, 2019, questionnaires were distributed manually to eighth-grade pupils who had younger siblings in second or third grade. The children who met the study’s inclusion criteria were given a letter asking their parents to participate in the study and providing the researchers’ contact details. Parents who indicated their willingness to participate (gave their informed consent) received a questionnaire, which they returned to the school a few days later. The response rate was 92%. The sampling method was manual rather than via an online questionnaire because a substantial portion of the Arab population, and particularly the Northern Bedouin population, has low digital literacy [59].

### Research tools

Prior to the quantitative study described in this paper, we conducted preliminary qualitative research using personal interviews with mothers of children at the targeted ages. The interviews focused on decision-making with respect to vaccinations [29]. Based on the results of this preliminary qualitative research and on validated questionnaires from the research literature focusing on different variables relevant to our research objectives [43, 60], we constructed a questionnaire (see Additional file 1) that was culturally adapted to the different subgroups in our study. After constructing the questionnaire, we calculated the Cronbach’s alpha value for items that appeared to be associated with measures of theoretical significance in order to validate each measure. Cronbach’s alpha is used to provide a measure of the internal consistency of a test or scale and is expressed as a number between 0 and 1. Internal consistency describes the extent to which all the test items measure the same concept or construct and hence reflects the inter-relatedness of the items within the test [61]. The questionnaire included **socio-demographic data** such as respondent’s age, number, age and sex of children, education, income, residential area, level of religiosity and ethnicity. It also included questions about **vaccination uptake** based on the mothers’ self-reports regarding the two relevant vaccinations recommended by the Ministry of Health: seasonal influenza and HPV. The statements in the first part of the questionnaire referred to variables related to vaccinations in general (called “general variables”). These included **attitudes toward vaccinations** (e.g., “All vaccinations recommended by the health authorities are safe”); **trust in doctors** (e.g., “When it comes to vaccinations, I trust my family doctor because he is the expert and knows more than I do”); **trust in the system** (e.g., “I trust the health system in Israel because of its high quality of care and service”); and **low health literacy**, referring to the extent to which the mothers think they are capable of seeking and reading information about vaccinations (e.g., “I don’t have time to look for information about vaccinations so I make do with what the medical team (nurse and doctor) tells me”).

The statements in the second part of the questionnaire focused on variables associated with each vaccination separately (called “specific variables”). For example, with respect to perceived risk, the questionnaire included statements about perceived risk of each disease and perceived risk of each vaccination (influenza and HPV, respectively). It also included statements related to perceptions regarding the inclusion of these vaccinations in the school-located vaccination program as a legitimizing factor for giving children these two vaccinations. Respondents were instructed to respond to each statement on a five-point Likert scale. The statements were grouped and defined as independent variables according to subject area (attitudes, trust, low health literacy and inclusion in the school vaccination program).

An examination of the correlations between all the independent variables yielded correlation coefficients less than 0.5. Therefore, we ran a multiple regression model. We also examined the associations between these variables and the dependent variable (uptake of the two types of vaccination: seasonal influenza and HPV) (see Table 1).

**Table 1:** Descriptive statistics: means, standard deviations and Cronbach’s alpha for the research variables

Variable	Mean	Standard deviation	Confidence interval (95%)		Cronbach alpha coefficient
			Lower boundary	Upper boundary	
**General attitudes regarding vaccinations**	3.70	0.76	3.64	3.76	0.60
**Trust in family doctor**	4.11	0.95	4.04	4.18	0.65
**Trust in health system**	3.77	1.07	3.70	3.85	0.72
**Low health literacy**	3.02	1.15	2.93	3.11	0.71
**Perceived risk of seasonal influenza**	2.81	1.01	2.73	2.88	0.60
**Perceived risk of cervical cancer**	2.27	0.88	2.20	2.33	0.40
**Perceived risk of HPV vaccination**	2.52	0..86	2.46	2.58	0.78
**Perceived risk of influenza vaccination**	2.90	0.92	2.83	2.96	0.80
**Including vaccination in school program**	3.40	1.10	3.32	3.48	0.60

### Reliability and validity

During questionnaire construction, the questions were formulated in Hebrew and translated into Arabic. They were then translated into Arabic a second time by a second translator to examine their cultural appropriateness and wording. After that, we conducted a pilot study among a sample of 80 participants to validate the content and check the wording to make sure it was culturally appropriate for the target population. After data collection and entry, quality control was applied to discover any errors in data entry. The quality control entailed examining the range of data for each question and generating distributions. In addition, the variables were examined for outliers [62] and tested to determine whether they met the assumption of normality.

### Data analysis

To compare vaccination uptake between the Jewish and Arab populations, we calculated the uptake rates for the two groups for the two vaccines. We used McNemar’s test to examine the significance of the differences between the uptake of the two vaccines in each of the subgroups. To identify the variables associated with mothers’ uptake of the two vaccines, we first conducted separate multiple logistic regressions according to type of vaccination, with uptake of the specific vaccine—HPV or influenza—as the dependent variable. Examination of the correlations between all the independent variables yielded coefficients that were all less than 0.5. Therefore, we were able to run a multiple regression model assuming no multicollinearity. We ran the multiple regression in two stages: In the first stage we ran the general variables and the specific variables in the multiple regression model to test the effect of each variable. In the second stage, we removed the variables that were not significant and ran the multiple regression again with the significant variables only to examine the exact effect of the variables on vaccine uptake. To examine the differences between the various subgroups with respect to variables associated with mothers’ uptake, first we used descriptive statistics and calculated the means of the variables among the different ethnic groups. Second, we conducted post-hoc testing for all the dependent variables: attitudes, trust in the system, trust in the doctor, low health literacy, school-located vaccination program, and risk perception of both vaccines. We then conducted a multiple comparison analysis using the Tukey correction to examine the significant differences between the various ethnic groups.

## Results

### Sample description

A total of 693 mothers participated in the study. The participants included mothers from almost the entire spectrum of the Israeli population. The Arab population was defined as the primary research population, while the national Jewish population (secular and religious/traditional groups) served for comparison purposes. Note that the ultra-Orthodox population was not included in the study. Table 2 shows their socio-demographic characteristics, followed by the mothers' education by ethnic groups and monthly income by ethnic groups (Tables 3 and 4 respectively).

**Table 2:** Mothers’ socio-demographic characteristics (*N* = 693)

Socio-demographic data	Category	(%)
**Age**	25–35	14.72
	36–40	41.56
	41–45	31.02
	46+	12.70
**Number of children**	2	13.71
	3	39.11
	4	28.86
	5	12.55
	6+	5.77
**Mother’s education**	Elementary	2.31
	Secondary	10.10
	Post-secondary	35.50
	Bachelor’s degree	24.39
	Master’s degree and higher	27.71
**Monthly Income (Relative to the average monthly income in Israel)**	Below 2500	2.05
	2500–4000	5.43
	4000–6500	14.81
	6500–8000	18.91
	8000–12,000	36.15
	Over 12,000	22.68
**Residential area**	City	39.02
	Village	56.07
	Moshav^a^	3.76
	Kibbutz^b^	1.16
**Level of Religiosity**	Secular	42.77
	Traditional	14.16
	Religious	42.12
**Ethnicity**	Northern Bedouin	14.72
	Muslim	24.53
	Christian	20.78
	Druse	17.17
	Jewish	22.08

^a^A moshav is a form of rural living unique to the State of Israel in which a group of residents live together in a joint financial arrangement. These residents are known as moshav members. Unlike the historical kibbutz framework, in the moshav the family is an independent financial unit operating in a framework of mutual assistance. Every moshav member is allocated a plot of land, which in most cases is used for agriculture [63]

^b^A kibbutz is a form of communal living unique to Zionism, the pre-state Yishuv period and the State of Israel, based on Zionist aspirations to resettle the Land of Israel as well as on the socialist values of human equality and of a joint economy and ideology. A kibbutz is usually a small locality with only a few hundred residents and supports itself through agriculture and industry [64]

**Table 3:** Mothers’ education by ethnic groups (*N* = 693)

Ethnic group	Elementary	Secondary	Post-secondary	Bachelor’s degree	Master’s degree and higher
Bedouin	20%	29%	28%	12%	11%
Muslim	1%	4%	44%	17%	34%
Christian	1%	3%	23%	28%	45%
Druse	1%	7%	51%	20%	21%
Jewish Religious	0%	9%	16%	51%	25%
Jewish Secular	2%	15%	29%	35%	20%

**Table 4:** Monthly income (in New Israeli Shekels) by ethnic groups (*N* = 693)

Monthly income (Relative to average monthly income in Israel)	Below 2500	2500–4000	4000–6500	6500–8000	8000–12,000	Over 12,000
Bedouin	8%	16%	29%	24%	24%	0%
Muslim	2%	5%	20%	25%	49%	0%
Christian	1%	4%	13%	17%	66%	0%
Druse	1%	5%	14%	23%	57%	0%
Jewish Religious	2%	2%	2%	11%	19%	65%
Jewish Secular	0%	1%	3%	8%	19%	61%

### Differences in uptake between Arab and Jewish populations

**Fig. 1:**
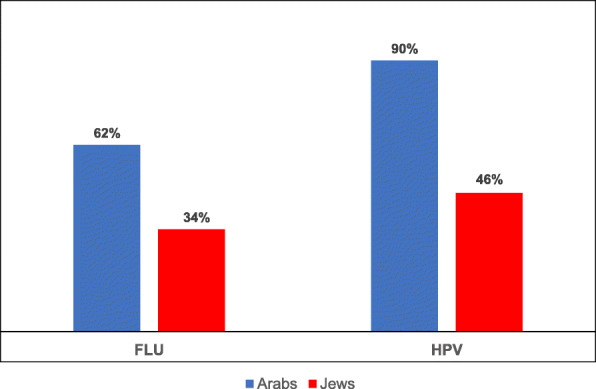
Vaccination uptake rates for Jews and Arabs

The research findings reveal differences in uptake of the two vaccinations between the Arab and Jewish populations, such that Arab mothers have a higher uptake rate for both vaccinations (HPV – 90%; influenza – 62%) than Jewish mothers (HPV – 46%; influenza – 34%) (Fig. 1).

The differences shown above are statistically analyzed in subsequent sections. Note that due to differences between the two vaccinations, we analyzed each of them separately. In addition, we found that in each case different factors influence vaccination uptake. Therefore, to examine the variables associated with mothers’ uptake of the two vaccinations, we computed two multiple logistic regression models and entered ethnicity as an independent variable in each. The models examined both general and specific variables associated with vaccine uptake.

Furthermore, McNemar’s test results reveal significant differences in uptake according to **type of vaccination**, showing that uptake of the HPV vaccination is significantly higher than uptake of the seasonal influenza vaccination in both populations: Arab (*p* < .0001) and Jewish (*p* = 0.0014).

### Variables specifically associated with mothers’ uptake of seasonal influenza vaccination

The first model for seasonal influenza vaccination included the general variables of ethnicity, attitudes, trust in the system, trust in the family doctor, school-located vaccination program and health literacy and the specific variables of vaccine risk perception and disease risk perception. On this model, the general variables of attitudes (*p* = 0.3286) and trust in family physician (*p* = 0.2715) were not significant. Therefore, to examine the precise effect of each variable on influenza vaccination uptake we decided to eliminate these two variables and run the multiple regression with significant variables only. Trust in the medical system was significant in the first model (*p* = 0.0199), but was no longer significant when entered into the reduced model. Therefore, the reduced model did not include this variable.

Table 5 shows the variables found to be significantly associated with uptake of the seasonal influenza vaccination.

**Table 5:** Variables significantly associated with uptake of the seasonal influenza vaccination

Odds ratio estimates, reduced model (only significant variables)		
Variables	OR	CI (95%)
**Ethnicity (Arabs vs. Jews)**	3.125	1.891–5.165
**Low health literacy**	1.429	1.166–1.751
**Inclusion in the school-located vaccination program**	1.839	1.555–2.175
**Perceived risk of influenza vaccination**	0.253	0.186–0.344
**Perceived risk of seasonal influenza disease**	1.751	1.370–2.238

The results show that the odds of flu vaccination uptake among Arab mothers is above three times the odds among Jewish mothers. Low health literacy is positively associated with Flu vaccination uptake, where for each unit for literacy index, the odds of the uptake increases by 43%. Inclusion in the school-located vaccination program is positively associated with Flu vaccination uptake, where for each unit for Inclusion in the school-located index, the odds of the uptake increases by 84%. Perceived risk of influenza vaccination is negatively associated with Flu vaccination uptake, where for each unit for Perceived risk of influenza vaccination index, the odds of the uptake decreases by 75%. Perceived risk of seasonal influenza disease is positively associated with Flu vaccination uptake, where for each unit for Perceived risk of seasonal influenza disease index, the odds of the uptake increases by 75%.

### Variables specifically associated with mothers’ uptake of HPV vaccination

The first model for HPV vaccination included the general variables of ethnicity, attitudes, trust in the system, trust in the family doctor, school-located vaccination program and health literacy and the specific variables of vaccine risk perception and disease risk perception. On this model, the general variables of attitudes (*p* = 0.3147), trust in family physician (*p* = 0.4995), low health literacy (*p* = 0.1324) and disease risk perception (*p* = 0.7337) were not found to be significant variables. Therefore, to examine the precise effect of each variable on HPV vaccination uptake we decided to eliminate these variables and to run the multiple regression with significant variables only.

Table 6 shows the variables found to be significantly associated with HPV vaccination uptake:

**Table 6:** Variables significantly associated with HPV vaccination uptake

Odds ratio estimates, reduced model (only significant variables)		
Variables	OR	CI (95%)
**Ethnicity**	6.404	3.793–10.811
**Trust in the health system**	0.739	0.575–0.950
**Inclusion in the school-located vaccination program**	1.507	1.163–1.953
**Perceived risk of HPV vaccination**	0.388	0. 263–0.571
**Sex of child (female vs. male)**	0.407	0.244–0.680

The results show that the odds of HPV vaccination uptake among Arab mothers is above six times the odds among Jewish mothers. Trust in the health system is negatively associated with HPV vaccination uptake, where for each unit for Trust in the health system index, the odds of the uptake decreases by 26%. Inclusion in the school-located vaccination program is positively associated with HPV vaccination uptake, where for each unit for Inclusion in the school-located index, the odds of the uptake increases by 51%. Perceived risk of HPV vaccination is negatively associated with HPV vaccination uptake, where for each unit for Perceived risk of HPV vaccination index, the odds of the uptake decreases by 61%. Besides, the odds of HPV vaccination uptake for female youth is 59% lower than the odds of uptake for male youth.

### Differences in mothers’ uptake of the two vaccination types by ethnic group

**Fig. 2:**
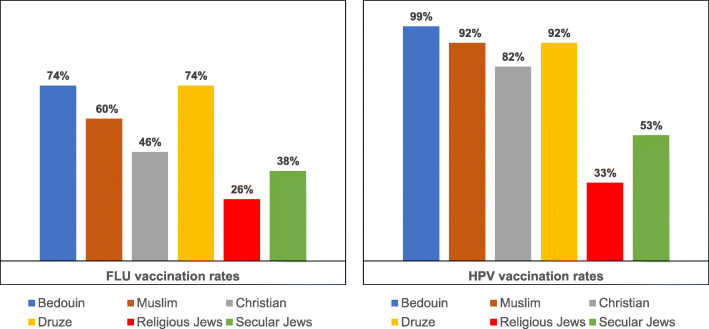
Interaction between vaccine type and ethnicity with respect to vaccination uptake

Examination of the ethnic subgroups reveals differences in mothers’ vaccination uptake. With respect to mothers’ uptake of the **seasonal influenza vaccination**, the highest uptake rates were found in the Northern Bedouin (74%) and Druse (74%) groups, followed by the Muslim group (60%). The lowest uptake rate in Arab society emerged among the Christians (46%). Moreover, secular Jewish mothers exhibited a lower uptake rate (38%) than any of the Arab groups, though higher than the religious/traditional Jewish mothers (26%), who exhibited the lowest uptake rate. With respect to **HPV vaccination**, the Northern Bedouin population exhibited the highest uptake rate (99%) of all the subgroups. The Druse population also exhibited a relatively high uptake rate (92%), as did the Muslim group (92%). Again the Christians exhibited the lowest uptake rate among the Arab society (82%). the secular Jewish mothers exhibited an HPV uptake rate of (53%), which was lower than all the Arab subgroups yet higher than the religious/traditional Jewish mothers (33%), who exhibited the lowest HPV vaccination uptake rate (see Fig. 2).

The results of the McNemar’s test (Table 7) show that in addition to differences between the ethnic groups with respect to uptake of the two vaccinations, each ethnic group (except for the religious Jewish group) exhibited significant differences in uptake according to **vaccination type:** HPV vs. seasonal influenza. The findings show that HPV vaccination uptake is significantly higher than seasonal influenza vaccination uptake in all the subgroups except for the religious Jewish group, where the difference is not significant.

**Table 7:** McNemar’s test *p*-value per ethnic group

Ethnicity group	*p*-value
Northern Bedouins	*p* < .0001
Muslims	*p* < .0001
Christians	*p* < .0001
Druse	*p* = 0.004
Secular Jews	*p* = 0.027
Religious Jews	*p* = 0.290

### Variables associated with vaccination uptake according to ethnic subgroup

**Fig. 3:**
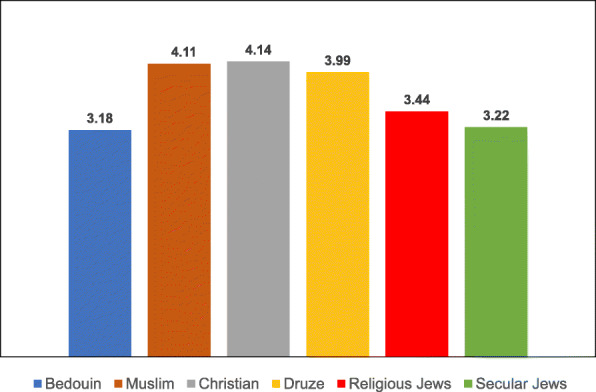
Means of trust the health system among different ethnic groups

**Fig. 4:**
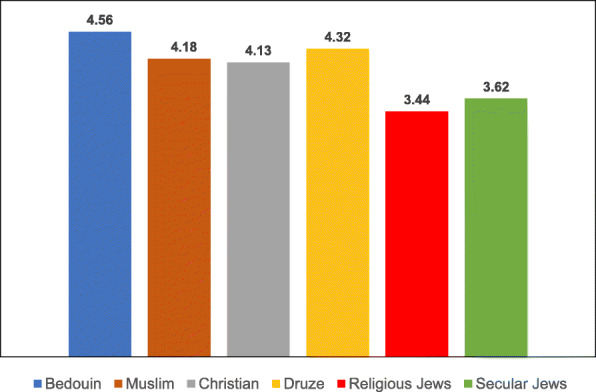
Means of trust the family doctor among different ethnic groups

**Fig. 5:**
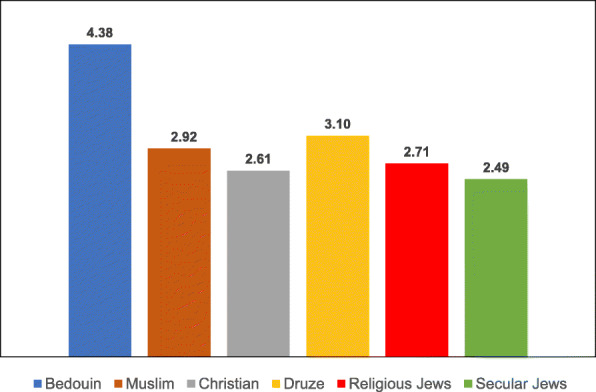
Means of low health literacy among different ethnic groups

**Fig. 6:**
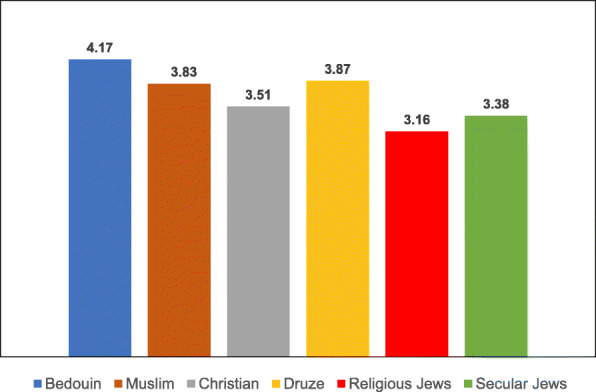
Means of attitudes toward vaccination among different ethnic groups

**Fig. 7:**
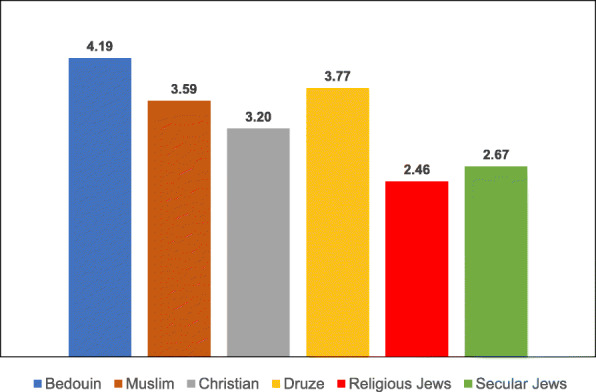
Means of including vaccination in school program as a legitimation to vaccination among different ethnic groups

**Fig. 8:**
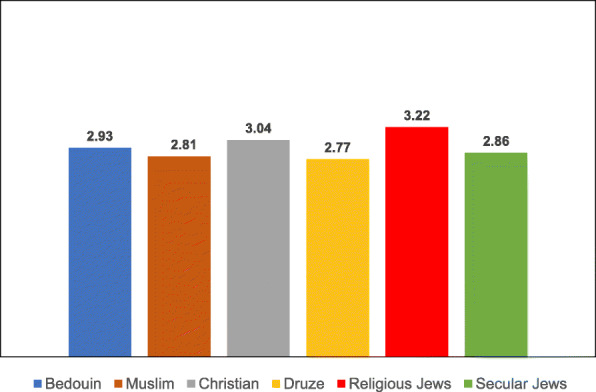
Means of perceived risk of influenza vaccination among different ethnic groups

**Fig. 9:**
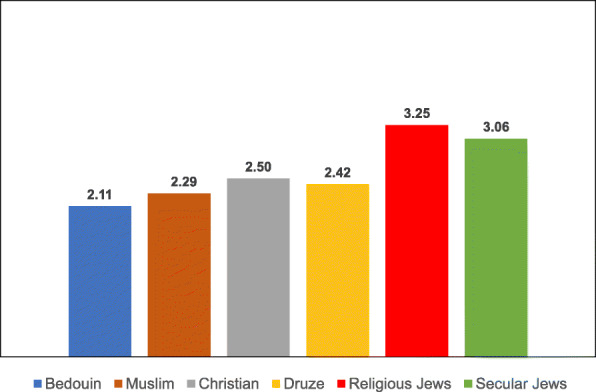
Means of perceived risk of HPV vaccination among different ethnic groups

Examination of the variables associated with uptake of the two vaccinations according to ethnic subgroup revealed differences in the means of both the general and the specific variables for each vaccination type, as illustrated in Tables 8, 9, 10, 11, 12, 13, 14 and the accompanying Figs. 3, 4, 5, 6, 7, 8, 9.

**Table 8:** Post-Hoc comparisons (Dependent Variable: Trust in the Health System) Tukey’s Studentized Range (HSD) test

Tukey grouping^a^	Mean	Ethnic group
A	4.1424	Christian
	4.1118	Muslim
	3.9916	Druse
B	3.4386	Religious Jews
	3.2228	Secular Jews
	3.1765	Bedouin

^a^Groups within the same letter are not significantly different

**Table 9:** Post-Hoc comparisons (Dependent Variable: Trust in Family Doctor) Tukey’s Studentized Range (HSD) test

Tukey grouping^a^		Mean	Ethnic group
	A	4.5637	Bedouin
B		4.3235	Druse
		4.1824	Muslim
		4.1285	Christian
	C	3.6188	Secular Jews
		3.4386	Religious Jews

^a^Groups within the same letter are not significantly different

**Table 10:** Post-Hoc comparisons (Dependent Variable: Low health literacy) Tukey’s Studentized Range (HSD) test

Tukey grouping^a^		Mean	Ethnic group
	A	4.3791	Bedouin
	B	3.0980	Druse
C		2.9216	Muslim
		2.7076	Religious Jews
		2.6088	Christian
		2.4851	Secular Jews

^a^Groups within the same letter are not significantly different

**Table 11:** Post-Hoc comparisons (Dependent Variable: Attitudes) Tukey’s Studentized Range (HSD) test

Tukey grouping^a^		Mean	Ethnic group
	A	4.1691	Bedouin
	B	3.8676	Druse
		3.8255	Muslim
	C	3.5139	Christian
D		3.3787	Secular Jews
		3.1623	Religious Jews

^a^Groups within the same letter are not significantly different

**Table 12:** Post-Hoc comparisons (Dependent Variable: Including vaccination in school program) Tukey’s Studentized Range (HSD) test

Tukey grouping^a^		Mean	Ethnic group
	A	4.1912	Bedouin
	B	3.7689	Druse
C		3.5912	Muslim
		3.2049	Christian
	D	2.6683	Secular Jews
		2.4561	Religious Jews

^a^Groups within the same letter are not significantly different

**Table 13:** Post-Hoc comparisons (Dependent Variable Perceived risk of influenza vaccination) Tukey’s Studentized Range (HSD) test

Tukey grouping^a^		Mean	Ethnic group
	A	3.2211	Religious Jews
B		3.0375	Christian
		2.9333	Bedouin
		2.8594	Secular Jews
		2.8068	Muslim
		2.7731	Druse

^a^Groups within the same letter are not significantly different

**Table 14:** Post-Hoc comparisons (Dependent Variable: Perceived risk of HPV vaccination) Tukey’s Studentized Range (HSD) test

Tukey grouping^a^		Mean	Ethnic group
	A	3.2515	Religious Jews
		3.0594	Secular Jews
	B	2.4954	Christian
C		2.4174	Druse
		2.2873	Muslim
		2.1144	Bedouin

^a^Groups within the same letter are not significantly different

The ANOVA for the dependent variable of trust in the health system revealed a significant difference between the different ethnic groups [F(5,687) = 24.13, *P* < 0.0001]. Multiple comparison analysis using the Tukey correction to examine the significant differences between the ethnic groups showed that Christian, Muslim and Druse women had a significantly higher level of trust in the health system than Jewish women (secular and religious) and Bedouin women.

The ANOVA for the dependent variable of trust in the family doctor revealed a significant difference between the ethnic groups [F(5,687) = 19.45, *P* < 0.0001]. The multiple comparison analysis using the Tukey correction showed that Bedouin women exhibited a significantly higher level of trust in the family doctor than all the other groups, except for Druse women. Moreover, the level of trust in the family doctor among Jewish women (secular and religious) was significantly lower than that of Arab women in all the ethnic groups.

The ANOVA for the dependent variable of Low health literacy revealed a significant difference between the ethnic groups [F(5,687) = 52.04, *P* < 0.0001]. Multiple comparison analysis using the Tukey correction showed that Bedouin women exhibited the highest level of Low health literacy, with a significant gap between them and all the other groups. Secular Jewish women exhibited the lowest level of Low health literacy, with a significant gap between them and three other groups—Bedouin, Druse and Muslim women.

The ANOVA for the dependent variable of general attitudes toward vaccination revealed a significant difference between the ethnic groups [F(5,687) = 24.53, *P* < 0.0001]. Multiple comparison analysis using the Tukey correction showed that Bedouin women exhibited the highest level of support for vaccinations, significantly higher than that of all the other groups. Druse and Muslim women were second in their level of support for vaccinations. The other groups—Christian and Jewish—exhibited a lower level of support for vaccination, with religious Jewish women exhibiting the lowest level of support, significantly lower than all the other groups with the exception of secular Jewish women.

The ANOVA for the dependent variable of vaccinations given at school revealed a significant difference between the ethnic groups [F(5,687) = 41.67, *P* < 0.0001]. Multiple comparison analysis using the Tukey correction showed that giving the vaccinations at school was the most significant factor for Bedouin women, significantly higher than for all the other groups. Jewish women (secular and religious) rated this factor as significantly lower than the Arab women from all the ethnic groups.

The ANOVA for the dependent variable of risk of seasonal influenza vaccine revealed a significant difference between the ethnic groups [F(5,687) = 2.81, *P* = 0.0161]. Multiple comparison analysis using the Tukey correction showed that perceived risk of the seasonal influenza vaccine was significantly higher among religious Jewish women than among Muslim and Druse women. No other significant differences in perceived risk were found among the other ethnic groups.

The ANOVA for the dependent variable of risk of seasonal HPV vaccine revealed a significant difference between the ethnic groups [F(5,687) = 28.4, *P* < 0.001]. Multiple comparison analysis using the Tukey correction showed that perceived risk of the HPV vaccination was significantly higher among Jewish women (secular and religious) than among Arab women in all the ethnic groups. Moreover, a significant difference in level of perceived risk of the HPV vaccination was found between Christian and Bedouin women, with Christian women perceiving the vaccination as riskier than Bedouin women.

For summary, Among the general variables, trust in the family doctor exhibited the highest mean in all the Arab ethnic groups. Similarly, trust in the medical system exhibited high means among the Arab mothers, with the exception of the Bedouin mothers, who exhibited a low level of trust in the medical system. The variable of vaccination inclusion in the school-located vaccination program exhibited relatively high means among all the Arab subgroups except for the Christians. The variable of low health literacy exhibited a low mean in all the ethnic groups except for the Northern Bedouin mothers, who reported major difficulties in searching for information about vaccinations. The Christian mothers had the highest literacy of all the Arab groups in searching for information, and the secular Jewish mothers had the highest literacy of all the subgroups.

With respect to **seasonal influenza vaccination**, Jewish mothers (and specifically religious as opposed to secular mothers) perceive the vaccination as more risky than Arab mothers from all the subgroups, except for Christian mothers, whose risk perceptions were equivalent to those of the secular Jewish mothers. With respect to the **HPV vaccination**, the highest risk perceptions were among the religious Jewish mothers and the lowest among the Northern Bedouin mothers.

## Discussion

This pioneering research study provides an in-depth examination of decision-making processes among subgroups in Arab society in Israel with respect to two vaccinations recently introduced to the school-located vaccination program: the HPV vaccination and the seasonal influenza vaccination. The study describes the variables associated with vaccination uptake among subgroups in Arab society as well as among certain segments of the Jewish population (secular and religious Jews). The study’s findings show that the variable of including the two vaccines in the school program is the primary variable influencing Arab mothers’ decision-making with respect to the HPV and seasonal influenza vaccinations. Vaccination inclusion in the school-located vaccination program encourages parents to vaccinate their children and increases the chances of vaccination uptake. With respect to framing strategies in health communication, vaccination inclusion in the school-based program grants the vaccination medical legitimacy, which also influences parental uptake [65]. These findings are in line with those of other studies showing various reasons for parental preference for vaccinating their children at school, among them lack of access to medical services, limited time to take children for vaccinations, inability to leave work for this purpose and more [66–68].

Perceived risk of the vaccination itself is also associated with mothers’ decision-making processes. This finding is compatible with other studies showing that parents decide not to vaccinate their children based on high risk perceptions related to a lack of trust in vaccination safety [14, 50, 69, 70]. Moreover, as in many studies, the findings of this study indicate that high risk perceptions about the illness are also associated with mothers’ uptake of the vaccinations. That is, the more risky mothers perceive an illness, the greater their chances to uptake a vaccination that prevents it [7, 8, 71–73].

The findings also show an association between trust in the medical system and decision-making with respect to the HPV vaccination. Other studies that examined decision-making for HPV vaccination among parents in Arab minority groups in Western countries also found this variable to be significant [74–76]. Yet despite high vaccination compliance, trust in the system is not very high even among the subgroups of Arab mothers. These findings can be explained by two factors: 1) Campaigns and explanatory materials designed to promote HPV vaccination in Arab society are not sufficiently transparent and lack cultural appropriateness [2, 65]; 2) The recommendations of doctors and nurses, considered by Bedouin society to be reliable sources of information, are not sufficiently explicit [29, 75, 77].

Contrary to the findings of many studies worldwide, the findings of the current study show that health literacy and difficulties in searching for information about vaccinations are positively associated with mothers’ decision-making. That is, the lower the mothers’ health literacy and the more difficulties they have in searching for information, the more likely they are to uptake vaccinations [78–81]. This high vaccination uptake rate despite low health literacy can be explained by the fact that these mothers do not search for impartial information about the vaccination but rather receive their information exclusively from the health system. Because they do not search for information, these mothers are not exposed to the scientific controversy surrounding the HPV vaccine [37–39] or to the questions raised about the effectiveness of the influenza vaccine [5, 51, 52]. Various studies have shown that minority groups usually have low health literacy, are less exposed to scientific controversies surrounding vaccinations and are less hesitant about vaccinations [2, 29, 82].

With respect to the sex of the child in the case of the HPV vaccination, the results of the current study are in line with other studies showing that the child’s gender plays a role in mothers’ decision-making regarding the HPV vaccination [27, 28, 50, 68, 82]. Indeed, the findings of the current study show that mothers are more likely to vaccinate boys than girls. In conservative societies, and particularly in Arab society, the matter of sexuality is generally taboo, particularly among women. Therefore, men in conservative societies are thought to be more likely to engage in frequent sexual relations than women, leading to the assumption that mothers are more likely to decide to give the HPV vaccination to their male children [83, 84].

With respect to the various population subgroups, the findings point to differences in mothers’ uptake rates. Specifically, the Northern Bedouin population emerged as the group with the highest vaccination uptake rate among all the Arab subgroups. We propose several explanations for this finding. First, it is possible to assume that these high vaccination rates derive from the fact that a significant portion of Northern Bedouin mothers are illiterate (more than 60%) [85]. Consequently, their health literacy is low and their ability to search for, read and analyze health information in general and information about vaccinations in particular is limited [86–88].

Several studies indicate that mothers with a high level of education have lower vaccination uptake rates due to their ability to search for information about vaccinations and make decisions based on facts and on “informed consent” [89]. Furthermore, the findings show that Bedouin mothers vaccinate their children despite their mistrust of the health system. It is reasonable to assume that the main and perhaps only information source for Northern Bedouin mothers is the Ministry of Health. Studies have shown that Bedouin mothers usually take institutional health directives seriously and implement them regardless of their level of trust [89, 90]. Moreover, despite this low level of trust in the system this group has a very high level of trust in doctors, making the family doctor’s recommendation a highly influential factor in mothers’ decision-making regarding vaccines. Thus they fully adopt the recommendations of the ministry or the doctor representing the health system [73, 91]. These findings contradict the findings of two studies conducted among the Bedouin population in the south of Israel, which showed that these Bedouins do not complete their children’s vaccination programs due to their lack of access to health services and lack of trust in the government [7, 73, 91]. It is important to note that the Bedouins living in the south, mainly those in unrecognized villages, have less convenient access to medical services than those living in the north examined in this research, whose superior access to medical services enables them to complete the vaccination programs.

The results of this study also show that the Druse population has the second highest uptake rates for both vaccinations. There are several ways to interpret this finding. Many members of the Druse population serve in the Israeli military forces. This fact, together with their high levels of trust in the government and its decision-makers [92], may explain their high uptake of various types of vaccinations. Moreover, a substantial portion of the Druse population identifies itself with the dominant Jewish national group rather than the minority Arab population. Over the years, a picture has emerged of Druse solidarity with the Zionist ethos, while the Druse simultaneously distance themselves from the Arab and Islamic themes resonant among the Israeli-Arab sector of society [86, 93]. Their desire to be part of the dominant Jewish population may lead to their similar or even higher vaccination uptake. Yet this interpretation may be qualified by the recently formulated basic law defining Israel as the Nation-State of the Jewish People,^1^ which may influence the reciprocal relations between the Druse and the State of Israel. Hence, future research is needed to verify this interpretation.

The research findings also indicate that Muslim mothers are third in uptake rate for the two vaccinations. Examination of the variables associated with vaccination uptake shows that the variable of inclusion in the school-located vaccination program is one of the most significant variables associated with Muslim mothers’ decision-making about the two vaccinations. It is possible to assume that including these vaccinations in the school program provides these mothers legitimization to vaccinate their children along with a convenient way to do so [7–9, 29].

With respect to the Christians, the final subgroup in the Arab population, the findings show that Christian mothers have the lowest vaccination uptake rate of all the Arab subgroups for both vaccinations. This finding can be explained by the fact that the Christian Arab population differs from the Muslim, Northern Bedouin and Druse groups in that they are more educated. Indeed, Christian society is marked by high socioeconomic status and a more modern lifestyle (for example, lower fertility rates) [94, 95]. Their relatively low vaccination uptake may be tied to their higher education and literacy levels, which enable Christian mothers to search for information from other sources [94–96]. Thus, the Christian mothers may be exposed to discourse on controversies surrounding vaccinations. The research findings also show that like the Christian mothers, secular Jewish mothers, who are in fifth place in vaccination uptake, vaccinate their children at lower rates than all the Arab subgroups. As indicated by the current research, due to their high educational level, their high level of knowledge about vaccinations and their more hesitant attitudes toward vaccinations, Jewish mothers tend not complete their children’s vaccination programs [2, 3, 7–9, 96, 97].

One of the more surprising findings of this study is related to uptake of the HPV vaccination among conservative population groups. The HPV vaccination is intended to prevent cervical cancer and genital warts caused by the human papillomavirus, which is transmitted through sexual relations. Arab society is considered to be a conservative and traditional society [29, 84], particularly in the context of sexuality and sexual relations prior to marriage, which are a social taboo [40–43, 98–100]. The findings of this study show that Arab mothers, without exception, vaccinate their children against the human papillomavirus at higher rates than Jewish mothers, despite the relationship between this vaccination and sexual activity. This finding can be explained by the lack of transparency that characterizes explanatory materials geared to increase awareness about the HPV vaccine among the Arab population. In another study in which we analyzed Arabic language explanatory materials issued by the Ministry of Health and the HMOs, we found that these materials did not refer to the sexual context of the vaccination, provided only partial information and were not culturally appropriate to Arab society [65]. Because Arab mothers are usually only exposed to information issued by the establishment and are unable to search for and process other information, it is reasonable to assume that they treat these materials as a reliable source of information and a basis for making decisions. Thus, promoting the HPV vaccine as preventing cancer serves to reframe the relationship between this vaccination and sexuality and increases the probability that the conservative Arab population will uptake the HPV vaccination. Religious Jewish society exhibits a cultural resemblance to Arab society in that it is also conservative and prohibits sexual relations before marriage. Nevertheless, the findings of this study show that the religious Jewish population differs from the Arab population with respect to vaccination uptake, as reflected in lower rates of HPV vaccination. These differences can be explained by the higher level of health literacy among religious Jewish mothers compared to Arab mothers, pointing to their greater ability to search for information and learn about the scientific controversy surrounding the vaccination and its association with sexuality, thus reducing their chances of HPV vaccination uptake [2, 29, 75].

This study was not designed to compare the Arab minority population in Israel to other Arab minorities worldwide regarding these two vaccinations. This issue should be the topic of future research.

This study has several limitations. First, the research was based on mothers’ self-reports regarding their vaccination uptake, increasing the chances of report bias. Second, the study focused on the Arab population as the main research population and the Jewish population as a comparison group and did not examine subgroups in Jewish society. We recommend extending the study to the Jewish population and examining the decision-making processes regarding these two vaccinations among different Jewish subgroups. Moreover, additional research is warranted to examine mothers’ decision-making with respect to various vaccinations, including identifying different variables that may have been associated with vaccination uptake over the years and detecting changes in vaccination trends, if any.

## Conclusions

This pioneering research study reveals variations in vaccination uptake among different population subgroups. The study points to the important influence of variables related to trust, literacy and legitimacy of school vaccination. It also shows that all Arabs cannot be lumped together as one monolithic group. Indeed, they exhibit major differences according to religion, education and access to information. Examining variables associated with uptake of the two vaccines can provide decision-makers an empirical basis for tailoring specific and appropriate interventions to each subgroup in order to achieve the highest vaccination uptake rate possible. The research also makes an important contribution to the literature on inequity in vaccination uptake as it exemplifies the variations within broad ethnic minority groups, which should be considered in policies and in practice. Moreover, media campaigns targeting the Arab population should be segmented to appeal to the various sub-groups according to their attitudes, needs and health literacy. The abilities and tools available to mothers must be reinforced so they can make intelligent decisions that are not based exclusively on trust in a third party such as the health or education system.

Vaccination hesitancy is on the rise worldwide, including in Jewish society in Israel. For this reason, it is important to take the public’s feelings of hesitancy into consideration and to build trust in the medical system. Note that this research was conducted before the coronavirus crisis in Israel, and it is likely that the crisis has affected vaccination uptake in Arab society as well. Future research is therefore needed to continue investigating these subgroups to examine the impact of COVID-19 on their attitudes toward vaccinations and their vaccination uptake.

## Supplementary Information

**Additional file 1.** Research questionnaire.

## Data Availability

Requests for more detailed information regarding the study should be addressed to the corresponding author.

## Abbreviations

HPV: Human papillomavirus
Flu: Influenza

## Footnotes

1 Basic Law: Israel as the Nation-State of the Jewish People, informally known as the Nation-State Bill or the Nationality Bill, anchors the national Jewish values of the State of Israel in a Basic Law, after many such values were already anchored in other laws. This Basic Law specifies the nature of the State of Israel as the nation-state of the Jewish people, the place where the Jewish people has a natural right to self-determination, a right that is exclusive to the Jewish people. The law also anchors the status of the state flag and state emblem and of “Hatikva” as the state anthem. It determines the use of the Hebrew calendar and the holidays of Israel and states that Hebrew is the official state language. The law also states that Jewish immigration is to be encouraged, that Jerusalem, complete and united, is the capital of Israel, and that Arabic is not a state language but has a special status in the state.
